# Cellular Immune Responses in Islet Xenograft Rejection

**DOI:** 10.3389/fimmu.2022.893985

**Published:** 2022-07-07

**Authors:** Min Hu, Wayne J. Hawthorne, Shounan Yi, Philip J. O’Connell

**Affiliations:** ^1^ Centre for Transplant and Renal Research, The Westmead Institute for Medical Research, Sydney, NSW, Australia; ^2^ The Faculty of Medicine and Health, University of Sydney, Sydney, NSW, Australia

**Keywords:** xenograft, islet (cell) transplantation, T cell, macrophage cell, transgenic pig, IBMIR

## Abstract

Porcine islets surviving the acute injury caused by humoral rejection and IBMIR will be subjected to cellular xenograft rejection, which is predominately mediated by CD4^+^ T cells and is characterised by significant infiltration of macrophages, B cells and T cells (CD4^+^ and CD8^+^). Overall, the response is different compared to the alloimmune response and more difficult to suppress. Activation of CD4^+^ T cells is both by direct and indirect antigen presentation. After activation they recruit macrophages and direct B cell responses. Although they are less important than CD4^+^ T cells in islet xenograft rejection, macrophages are believed to be a major effector cell in this response. Rodent studies have shown that xenoantigen-primed and CD4^+^ T cell-activated macrophages were capable of recognition and rejection of pancreatic islet xenografts, and they destroyed a graft *via* the secretion of various proinflammatory mediators, including TNF-α, reactive oxygen and nitrogen species, and complement factors. B cells are an important mediator of islet xenograft rejection *via* xenoantigen presentation, priming effector T cells and producing xenospecific antibodies. Depletion and/or inhibition of B cells combined with suppressing T cells has been suggested as a promising strategy for induction of xeno-donor-specific T- and B-cell tolerance in islet xenotransplantation. Thus, strategies that expand the influence of regulatory T cells and inhibit and/or reduce macrophage and B cell responses are required for use in combination with clinical applicable immunosuppressive agents to achieve effective suppression of the T cell-initiated xenograft response.

## Introduction

Reversal of established type 1 diabetes (T1D) requires beta cell replacement and immunosuppressive treatment to eliminate immune responses against them. Currently, beta-cell replacement is limited by supply and the need for long-term immunosuppression. Existing islet allotransplant programs have demonstrated the proof of concept and successful transplantation leads to normal blood glucose control (1, 2). However, it is not a complete answer to these challenges, which include, limited cell numbers, problems with islet isolation, and complications from immunosuppression. Future cell-based therapies will require the development of new technologies including immune tolerance, stem-cell therapies, xenografts and cell re-programming. All of these technologies have advantages and disadvantages. For islet xenotransplantation, the greatest challenge is overcoming the strong immune response. Its major advantages are the fact that it is a stable committed beta cell that does not require reprogramming and it is readily amenable to gene editing technologies to overcome the issues with rejection. This review will focus on the major cellular immune responses to islet xenografts and will touch upon potential interventions to overcome these. It is important that we understand the mechanisms of islet xenograft rejection, so that we can better utilise one of the major advantages of xenotransplantation – the ability to genetically modify the donor in order to avoid the recipient immune response.

## Innate Mechanisms of Islet Xenograft Rejection

**Figure 1:**
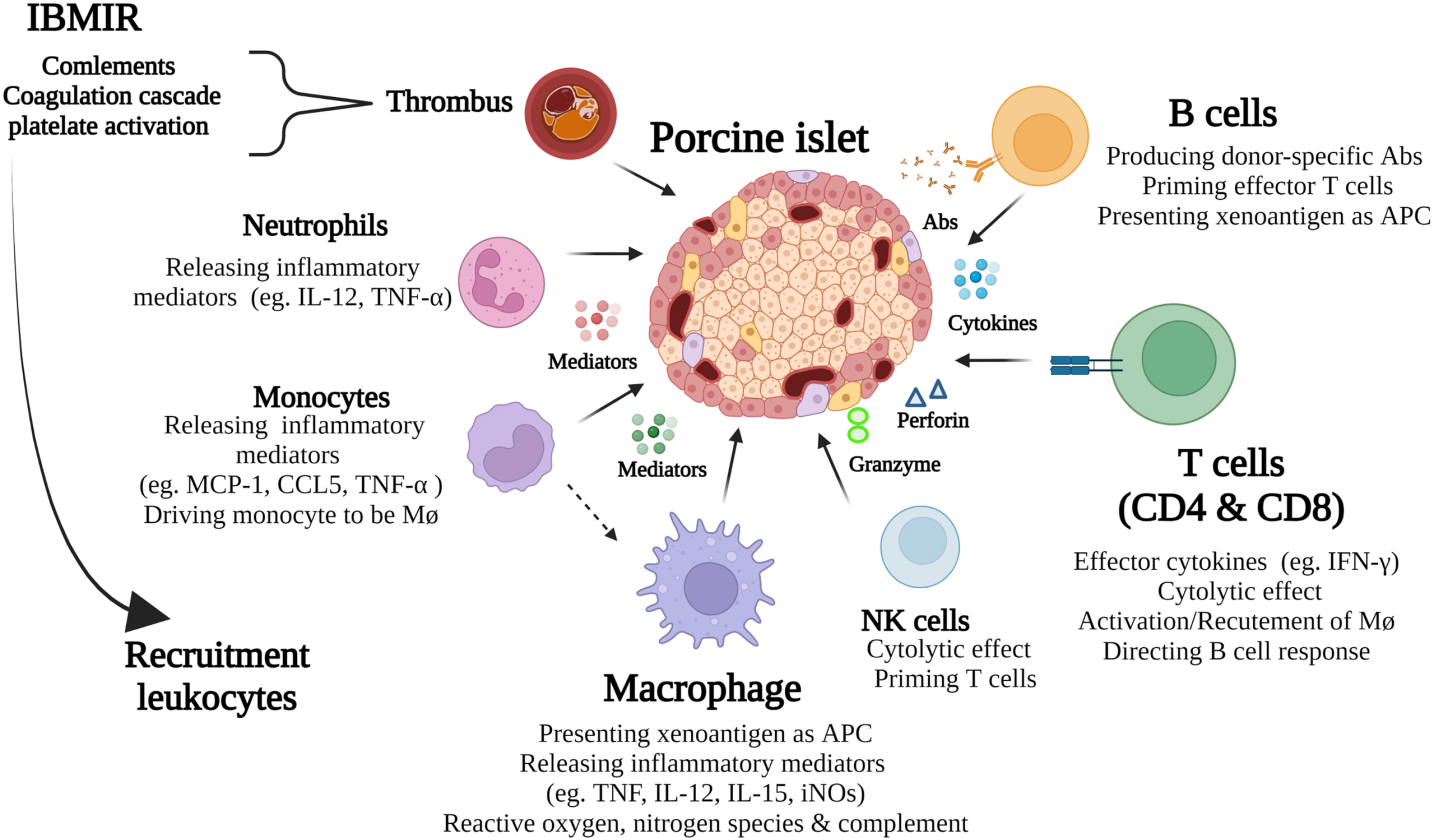
Schematic diagram of key immune responses in porcine islet xenograft rejection. Immediate blood mediated immune response (IBMIR) is an innate immune response that in turn amplifies the ongoing cognate response by further recruitment of leukocytes to the grafts. These include neutrophils, monocytes, macrophages, NK, B, and T cells. The ensuing adaptive cellular response, mediated by T cells, macrophages and B cells, plays a major role in islet xenograft rejection. As well as being important effector cells both macrophages and B cells serve as APC that activate T cells.

Islet xenografts are thought to avoid the major problems of hyperacute and delayed xenograft rejection. Primarily this is because in rodent models, the islet grafts are placed under the renal capsule, where they undergo neovascularisation *via* the recipients’ vascular supply. However, in clinical transplantation, islets are transfused into the portal circulation where they have direct exposure to human blood. In clinical islet allotransplantation, it is estimated that 50% of islets are lost to an innate thrombotic response called immediate blood mediated immune response (IBMIR) (3, 4). IBMIR is characterized by an initial activation of the coagulation and complement systems with rapid activation and binding of platelets and the recruitment and infiltration of leukocytes (5). Human and pig islets both express tissue factor, a potent activator of the extrinsic pathway of coagulation and inhibiting its expression with a monoclonal antibody suppressed the response *in vitro* (6). Apart from the amplification and propagation of coagulation, thrombin is also a critical molecule for the recruitment of inflammatory cells such as activated platelets, monocytes and neutrophils (**Figure 1**). These cells secrete IFN-γ, IL-12 and TNF, as well as other chemokines and cytokines which in turn amplify the cognate immune response to the graft. Other important initiators of IBMIR include preformed antibody and complement. Porcine neonatal islet cell clusters (NICC) express the oligosaccharides galactose α1-3 galactose, N-glycolylneuraminic acid as well as glycans produced by β1,4 N-acetylgalactosaminyl transferase (7). Humans with preformed antibodies to these antigens bind immediately to NICC leading to complement activation *via* the classical pathway. In the absence of antibodies, pig islets have been shown to activate complement *via* the alternate pathway. Once activated, C3a and C5a lead to further recruitment of neutrophils and monocytes and the formation of the C5b-9 complex leads to cell lysis (8). The hyperacute rejection and IBMIR have been reported to be overcome by gene editing donor islets and using certain immunosuppressive regimens in nonhuman primate (NHP) porcine islet transplanted recipients (9–11). In a NHP model of islet xenotransplantation, IBMIR was overcome by infusing intraportally a large number (25000/Kg) of cultured wild-type adult porcine islets under immunosuppression with rapamycin and CTLA4Ig (12).

## Cell-Mediated Xenogeneic Immunity in Porcine Islet Xenotransplantation

Porcine islet xenografts that survive IBMIR are subjected to a cognate immune response that is amplified by IBMR related inflammation and recruitment of leukocytes to the graft leading to a cell-mediated immune response (**Figure 1**). The cellular responses to porcine islet xenografts are mediated by both innate and adoptive immune cells and are different compared with alloimmune responses. Although the innate immunity mediated by natural killer (NK) cells and neutrophils are thought to be involved in porcine islet xenograft rejection, this review will focus on cell-mediated immunity by T cells, B cells, and macrophages in porcine islet xenotransplantation.

## Macrophage-Mediated Cellular Immunoresponse in Islet Xenotranplantation

Macrophages are a key effector cell of the innate immune system. In xenotransplantation, they exert phagocytic action and modulate adaptive immunity by contributing to cell recruitment and antigen presentation (13, 14). Upon contact with xenogeneic cells, macrophages present xenoantigens to generate Th1 and Th17 cells in acquired immunity (14), and allow the recruitment of both CD4^+^ and CD8^+^ T cells into the islet xenograft (15). Macrophages also play important roles in pro-inflammatory and oxidative events that occur in xenotransplantation (16, 17). They initiate tissue damage mediated by reactive nitrogen and oxygen species (18, 19), and promote inflammation by recruiting and activating neutrophils (20). The macrophage-involved local innate immune response stimulates T cell infiltration and in turn, the xenograft infiltrating CD4^+^ T cells mediate optimal macrophage activation (15, 21), possibly *via* the IFN-γ pathway (22, 23), thereby leading macrophages to act as direct effector cells in xenograft rejection (15, 21). In rodents, macrophages are the earliest infiltrating cell population within islet xenografts (24). We and others have shown that T-cell-initiated islet xenograft rejection was accompanied by a large accumulation of macrophages in the rejecting grafts (21, 25), and that CD4^+^ T cell-activated macrophages harvested from porcine islet recipient NOD-SCID mice with rejecting grafts were capable of both recognition and rejection of pancreatic islet xenografts when transferred to secondary NOD-SCID islet xenograft recipients in the absence of T cells (21). Moreover, surface accumulation and overgrowth composed of macrophages is also reported to be one of the key mediators inducing functional failure of encapsulated xenogeneic rat or porcine islets (26–28). Treatment of recipient C57BL/6 (B6) mice with macrophage depleting agents, gadolinium chloride (GdCl) or liposome-encapsulated dichloromethylene diphosphonate (Lip-Cl2MDP), prolonged survival of their human or porcine islet xenografts, with decreased xenograft infiltrating macrophages when compared with untreated controls (25, 29).

Once activated and attracted to the xenograft, macrophages are capable of graft destruction mediated *via* the secretion of various proinflammatory mediators, including TNF-α, reactive oxygen, nitrogen species and complement (14, 21). Indeed, upregulated expression of inflammatory mediators and cytolytic molecules such as IL-12, IL-15, TNF-α, and iNOS were identified in the graft-infiltrating macrophages of rejecting porcine islet recipient NOD-SCID mice transferred with activated macrophages (without T cells) from mice with rejecting porcine islet xenografts (21). This macrophage-mediated cytotoxic process was found to be surprisingly xenoantigen specific. When syngeneic mouse and pig islets were admixed and placed under the kidney capsule of recipient B6 mice, only the pig islets were destroyed (30). Furthermore, adoptive transfer with activated macrophages did not affect the normalized blood glucose levels in NOD-SCID mice transplanted with admixed mouse islet allografts and porcine islet xenografts under the left kidney capsule, and islet xenografts alone under the right kidney capsule until left nephrectomy 5 weeks post-macrophage transfer. Insulin-positive mouse- but not porcine-islets were detected in the admixed grafts with no visible graft infiltrating macrophages, thereby providing direct evidence of porcine islet xenograft specific rejection by activated macrophages (31).

Chemokines are likely to be involved in attracting macrophages to the graft site. Monocyte chemoattractant protein-1 (MCP-1) was shown to be important in attracting macrophages into the xenoislets. In an *in vitro* setting, adult porcine islets (APIs) cultured for 1, 4, 8 and 11 days post-isolation, expressed mRNA for MCP-1, IL-1β and TNF-α. Supernatants harvested from the APIs culture induced migration of human monocytes, which was significantly blocked by an anti-human MCP-1 antibody. Thus, it suggests that MCP-1 secreted by APIs may attract monocytes into the site of islet xenografts; monocytes which upon transformation into macrophages then executed islet xenograft rejection (32). Intragraft gene expression profiles obtained from both pig-to-nonhuman primate and -to-mouse islet xenotransplantation models showed that upregulated MCP-1 expression within the grafts correlated with early macrophage infiltration (33–35). Transplantation of porcine islets to CCR2 deficient mice (lacking the major receptor for MCP-1) delayed intragraft recruitment of macrophages and CD4^+^ T cells, but ultimately graft rejection occurred (33). Collectively, these studies identify MCP-1 as an important molecule in regulation of macrophage and/or CD4 T cell infiltration to xenograft sites *via* the CCR2 signalling pathway (33). In addition to confirming MCP-1, our studies have demonstrated that other signalling pathways may be required for macrophage recruitment and activation in the islet xenografts (36–38). While macrophages isolated from rejecting porcine islet xenografts of wildtype B6 mice demonstrated upregulated expression of macrophage activation markers, as well as CCR5 and CCR2 genes, and caused pig islet xenograft destruction 8 days after adoptive transfer to NOD-SCID recipients, the graft infiltrating macrophages from rejecting CCR5-/- recipients showed impaired macrophage activation when compared to control B6 recipients, and transfer of these macrophages did not result in xenograft destruction in NOD-SCID recipients until day 16 after transfer. Analysis of graft infiltrating macrophages from these rejecting NOD-SCID mice showed an impaired activation phenotype, indicating the importance of CCR5 in both the activation and recruitment of macrophages to rejecting islet xenografts (36). In pig-to-primate islet xenotransplantation, the expression of mRNA CXCR3, interferon-inducible protein 10 (IP-10), and monokine induced by IFN-*γ* (Mig) increased significantly from 12 to 72 hours in NHP recipients after intraportal infusion of pig islet and was associated with predominantly neutrophil and macrophage infiltration in the first 72 h hours after transplantation (35). In specially designed tracking studies, activated macrophages were shown to track to and reject recently transplanted but not established FPP xenografts with upregulated gene expression for MCP-1, RANTES, MIP-1alpha and MIP-1beta detected in recently transplanted but not in established xenografts. This suggests that graft-mediated pro-inflammatory signals were important for macrophage recruitment. Moreover, when exposed to porcine islets, macrophages expressed higher levels of TLR genes, compared with those exposed to allografts regardless of the level of their activation, demonstrating that TLRs may be involved in specific graft recognition by macrophages (37).

The macrophage-involved islet xenograft rejection has also been shown in NHP studies, where T cells preceded the influx of macrophages into the graft after intraportal porcine islet injection (39), and porcine islet graft failure that occurred in recipient non-immunosuppressed NHP was associated with massive intra-islet infiltration by CD4+ and CD8+ T cells and macrophages (40, 41). Moreover, in a dual islet transplant NHP model, α1,3-galactosyltransferase knockout (GalT KO)-derived NICC and rhesus islet allografts (AIs) were intraportally infused into rhesus monkey recipients. At day 7 after transplantation, highly specific macrophage infiltration and IgM accumulation were detected in islet xenografts compared with their allograft counterparts, indicating an early augmented and specific macrophage and antibody response towards the xenografts (42).

In xenotransplantation, cross-species incompatibility between inhibitory signalling ligands and their receptors can occur. Such is the case with CD47-SIRPα signalling which can lead to macrophage activation (19). CD47, a marker of self on most cells, binds to SIRPα on macrophgages thereby preventing the clearance of cells by phagocytosis. SIRPα is expressed on macrophages and dendritic cells (DCs) and recognizes CD47. The lack of any functional interaction between porcine CD47 on the cell surface of xenografts with the human species-specific macrophage inhibitory receptor, SIRPα, makes porcine cells more susceptible to macrophage-mediated damage (19). *In vitro* studies using porcine red blood cells, endothelial cells, and a kidney cell line have shown that cross-species incompatibility of SIRPα-CD47 interactions contributes to the rejection of xenogeneic target cells by macrophages, and transgenic expression of human CD47 on porcine cells significantly reduced the human macrophage-mediated phagocytosis of xenogeneic target cells *in vitro* (19) (43–45). The suppression of macrophage activation by the transgenic expression of hCD47 on the graft has been confirmed in nonhuman primate xenotransplantation models. These studies showed that the transgenic expression of human CD47 promotes the engraftment and survival of skin xenografts (46) and porcine hematopoietic progenitor cells (47) in a pig to baboon xenotransplantation model. Taken together, these data suggest that macrophage activation and phagocytic activity resulting from the cross-species molecular incompatibility of SIRPα-CD47 interactions may also contribute to porcine pancreatic islet xenograft rejection.

Strategies to inhibit the macrophage-mediated immune response in xenotransplantation have been proposed in a number of studies. *In vitro* coculture of pig stimulator cells with human CD14^+^ macrophages and CD4^+^CD25^-^ T cells in the presence of autologous CD4^+^CD25^+^ Tregs has been reported to lead to substantially decreased expression of macrophage activation markers and reduced capacity of macrophages to stimulate proliferation of responder T cells. This indicates that Tregs were capable of suppressing xenoantigen-primed and CD4^+^ T-cell-mediated macrophage activation and antigen-presenting cell function (48), thereby supporting Tregs as a potential immunotherapy to inhibit macrophage-mediated xenograft rejection in islet xenotransplantation. Targeting chemokines that inhibit the activation and recruitment of macrophages to the graft may also be an attractive option, which could prevent macrophage migration without significantly altering normal immune cell function. Other strategies include modifying chemokines involved in the recruitment of macrophages so that they act as specific antagonists devoid of non-specific effects (35). This approach has been investigated in the treatment of asthma, showing reduced allergic airway inflammation (49). Human CD47 transgenic pigs have been generated, and prolonged survival of human CD47 pig-derived xenografts in NHP has been achieved by reducing macrophage activation (46, 50, 51). Generating pigs with human leukocyte antigens-E (HLA-E) (52) or human leukocyte antigens-G (HLA-G) (53) has also been proposed as an additional option to inhibit human macrophage-mediated porcine islet xenograft rejection. The overexpression of CD200 on porcine cells suppressed xenogeneic activation of human macrophages and prolonged porcine xenograft survival in humanised mice (55).

Current immunosuppressive regimens applied in islet xenotransplantation primarily target adaptive T-cell immunity, and while they have improved short-term outcomes they have failed to achieve long-term xenograft survival. Thus, it raises the possibility that immune processes, such as macrophage-mediated immune-responses, are additional players that promote xenograft failure. Therapies targeting both T cell and macrophage activation and their interaction may be required to make a more substantive impact on long-term islet xenograft survival.

### The T Cell Response to Porcine Islet Xenografts

T cells plays a central role in initiating the cellular immunoresponse in islet xenotransplantation (54). The T cell response to xenografts is quantitatively and qualitatively different to the alloimmune response or autoimmunity where there are at least two distinct pathways for antigen presentation, i.e. the direct and indirect pathways (55). The direct pathway of antigen presentation, the major initial T cell activation pathway in allorecognition, is characterized by the recipient’s T cells recognizing intact allogeneic major histocompatibility complex (MHC) molecules on the surface of donor antigen present cells (APCs). The indirect pathway involves the uptake of donor’s allogeneic HLA peptides by the recipient’s own APCs and their presentation to effector T cells by self-MHC molecules. In alloimmune rejection, there is a major role for CD8^+^ T cells (56). Whereas in islet xenotransplantation the predominant cell involved is the CD4^+^ T cell (22, 57–59). Depletion of CD4^+^ T cells by anti-CD4 monoclonal antibody prolonged ICCs xenograft survival (58). In a humanized ICCs xenotransplantated mouse model, adoptive transfer of *in vitro* porcine PBMC-stimulated human PBMC into immunodeficient mice led to acute cellular rejection of ICCs with CD4^+^ T cells observed to be the first and major cell type infiltrating rejecting grafts (59). The central role of CD4^+^ T cells in co-ordinating the cognate response has been further confirmed in SCID mouse models where adoptive transfer of small numbers of CD4^+^ T cells lead to robust xenograft rejection (22, 57) and the CD4^+^ T cell response to porcine islets has been shown to be predominantly *via* indirect recognition (58, 60). The role for CD8^+^ cells is less clear. CD8^+^ T cells can lead to xenograft rejection *in vivo* although the time course is prolonged. This was interpreted as an inability by CD8^+^ T cells to directly recognise porcine class I MHC (61). Whilst this may be true in rodent models it may not hold for humans where it has been shown that T cells are capable of direct recognition of porcine MHC (62, 63). In a series of elegant mixed lymphocyte response assays it has been shown that human T cells interact directly with porcine MHC at a precursor frequency similar to alloimmune responses (62, 63). However the precursor frequency for the indirect response (i.e. presentation of porcine antigen *via* host antigen presenting cells) was substantially larger than in the allo-immune response due the large molecular incompatibilities between humans and pigs (64–66). These differences in precursor cell frequency and antigen presentation in turn leads to a different and stronger T cell response than one would anticipate for islet allotransplantation.

In NHP models of islet xenotransplantation islets are infused intraportally as is the case in clinical islet allotransplantation. Those islets that escape IBMIR undergo T cell-mediated acute cellular rejection where both CD4^+^ and CD8^+^ can be seen infiltrating the islet xenografts (39–41). As in murine studies this was predominately *via* the indirect pathway of antigen presentation (40, 41). Interestingly, there is strong indirect data supporting an important role for CD8^+^ T cells in porcine islet xenograft rejection in NHP. Using three different immunosuppressive regimens, Chung et al. measured the absolute number and ratio of T‐cell subsets *via* flow cytometry in porcine xenograft recipients and demonstrated that the ratio of CD4^+^ versus CD8^+^ T cells was significantly reduced due to an increase in CD8^+^ effector memory cells. In their models graft rejection was associated with a larger CD8^+^ T cell count suggesting CD4^+^/CD8^+^ ratios could be used as a surrogate marker to predict the graft fate in pig-to-NHP islet xenotransplantation (67).

The T cell mediated effector mechanisms seen in NHP models of porcine islet xenotransplantation are extensive and there are both qualitative and quantitative differences with the alloimmune response. Once activated, CD4^+^ T cells lead to the accumulation and activation of NK cells and macrophages *via* an IFN-γ dependent mechanism (22). As in rodent models, activated macrophages are seen to infiltrate into islet xenografts in NHP (40, 41). Apart from the recruitment of monocytes and NK cells, there is cytokine production and strong T cell-directed B cell responses leading to the production of anti-graft antibodies (68). The implications of these studies for the clinical implementation of islet xenotransplanation is that the potential for, and strength of, the T cell response will be greater and this is further amplified by the greater impact of IBMIR and innate immune activation. Unless this is modified by genetic modification of the donor, stronger and broader immunosuppression will be required.

## B Cell-Mediated Immune Response in Islet Xenotransplantation

B cells are now recognized as a key mediator of both acute and chronic allograft rejection through both antibody-mediated and antibody-independent functions, including generation of humoral responses, antigen presentation, priming of effector cells, and primary cytokine production (69–73). The B cell-mediated immunoresponse in islet xenotransplantation has been shown in animal studies. In fish islet to mouse xenotransplantation models, diabetic NOD recipient mice rejected their encapsulated fish islet xenografts on day 11 ± 4 with a peritoneal infiltrate of macrophages, eosinophils, B cells, occasional neutrophils, but few T cells, and murine IgG was seen attached to rejecting fish islets within the capsules of non-immunosuppressed mice (68, 74). In contrast, encapsulated fish xenoislets survival was significantly prolonged to 29 days when B-cell knockout NOD mice (NOD B-cell KO mice) were used as recipients (74). The involvement of B cells in encapsulated islet xenograft rejection was further confirmed by transplantation of microencapsulated NPIs into streptozotocin-induced diabetic immune-competent B6 and immune-deficient B6 rag-/- mice, showing that B6 mice rejected encapsulated NPIs within 14 days post-transplantation with a cellular overgrowth of CD4^+^ T cells, B cells and macrophages on the surface of encapsulated NPIs as well as mouse IgG antibody and complement detected within the microcapsules (75). In contrast, B6 rag-/- recipients maintained normoglycemia for up to 100 days post-transplantation with no cellular overgrowth on the surface of their microencapsulated NPIs (75). In a naked islet xenotransplantation mouse model, non-immunosuppressed prediabetic NOD mice immunosuppressed with anti-T cell monoclonal antibodies alone prolonged their xenograft survival to 80 days, but rejection occurred despite marked depletion of T cells. Interestingly, the addition of cyclophosphamide, a powerful anti-B cell agent led prevented late rejection, and prolonged survival for at least 112 days (76), further supporting the importance of the B cell-mediated immune response in islet xenotransplantation. Moreover, it was shown that treating B6 mouse recipients of human islets with anti-CD45RB mAb in combination with anti-CD20 mAb produced indefinite graft survival whereas only 26% of grafts survived in mice treated with anti-CD45RB mAb alone. When B6μMT-/- mice were used as recipients 89% of xenografts survived long term after anti-CD45RB-treatment in this B cell deficient mouse model, further demonstrating the importance of B cells in the xenograft rejection response (77). Indeed, targeting B cells combined with suppressing T cells has been suggested as a promising strategy for induction of xeno-donor-specific tolerance in islet xenotransplantation (78–80). This is supported in other models such as combined T cell inactivation by infusion with donor apoptotic 1-ethyl-3-(3′-dimethylaminopropyl)-carbodiimide-treated splenocytes (ECDI-SP) in combination with depletion of B cells with anti-CD20 mAb led to the indefinite survival of rat islet xenografts with inhibition of donor specific antibody production. Moreover, this combined treatment synergistically suppressed xeno-donor-specific T-cell priming as well as memory T-cell generation, and after initial depletion, the recovered B cells in long-term tolerized mice exhibited xeno-donor-specific hypo-responsiveness (80). However, when extended to a pig islet to B6 mouse xenograft model, long term tolerance could not be reproduced although graft survival was prolonged. Ultimately, grafts were lost at the time of B Cell reconstitution from a combined T and B cell anti-donor response, thereby confirming the involvement of B cells in both early and late islet xenograft response (79). Similar results have been seen in humanized mice where untreated recipient mice rejected their porcine islet xenografts as early as 14 days with heavy deposition of human IgM, and a significant graft infiltrate by human CD20+ B cells, CD3+ T cells, and CD68+ macrophages. Treatment with a combination of ECDI-SP, rituximab (a chimeric monoclonal antibody against CD20) and rapamycin prolonged porcine islet xenograft survival beyond 60 days with minimal infiltration of human immune cells or human IgM deposition (78). Again confirming the importance of T cell activated B cells in the xeno-immune response.

## The Role of Regulatory T Cells in T Cell Mediated Islet Xenograft Rejection

Historically, regulatory T cells **(**Tregs) were defined by their suppression of the immune response after activation by antigen (81) which was elegantly demonstrated in experimental models of transplantation tolerance (82). Subsequently, Tregs were characterized as CD4^+^CD25^+^ T cells that were identified to have the capacity of preventing autoimmunity and were responsible for the maintenance of transplantation tolerance in animal models (83–85). Later, forkhead/winged-helix transcription factor 3 (Foxp3) was identified as the key transcription factor which characterized this lineage of thymically derived Tregs (86, 87), and absence or mutation of the Foxp3 gene leas to severe immune dysregulation in both mice and humans (86, 88). Under certain circumstances, Foxp3^+^ Tregs can be induced peripherally from conventional non-Foxp3 CD4^+^ T cells by antigenic stimulation in the presence of TGF-β and IL-2 (89). There is a strong correlation between CD4^+^CD25^+high^CD127^-/low^ T cells and Foxp3 expression for human Tregs (90, 91). Therefore, human Foxp3^+^ Tregs are generally defined as CD4^+^CD25^+high^CD127^-/low^ cells. Several subsets of Tregs exist such as CD4^+^ type 1 Treg (Tr1) (92), regulatory γδ T cells (93), and CD8^+^ Tregs (94). Here we focus the discussion on the role of CD4^+^Foxp3^+^ Tregs in islet xenotransplantation.

In islet xenotransplantation, CD4^+^Foxp3^+^ Tregs play an important role in moderating the T cell mediated response for preventing rejection and the promotion of transplantation tolerance (95, 96). There are many immunomodulatory approaches to induce islet xenograft tolerance and costimulation blockade has been investegated intensively in animal models. Blocking the B7-CD28/CTLA-4 pathway by CTLA-4 Fc (a fusion protein consisting of mouse CTLA-4 and immunoglobulin Fc region) and/or the CD40-CD40L pathway by anti-CD154 monoclonal antibodies (mAb) prolonged islet xenograft survival or induced tolerance in human-to-rodent (97), rat-to-mouse (98, 99) and pig-to-mouse (100–104) models of islet xenotransplantation. CD4^+^Foxp3^+^ Tregs has been shown to play a critical role in these models (99, 103, 105). In a murine model of porcine NICC xenotransplant tolerance, short-term treatment with CTLA4-Fc and anti-154 mAb led to a clonal expansion of CD4^+^Foxp3^+^ Tregs in the spleen and tolerant xenografts (103). These CD4^+^Foxp3^+^ Tregs demonstrated the capacity to transfer dominant tolerance, expressing high levels of IL-10 and showed potent suppressive function suggesting donor antigen specificity (103). Depletion of Foxp3^+^ Tregs in recipient mice abolished pig islet xenograft tolerance, thereby confirming the essential role of CD4^+^Foxp3^+^ in this model (103).

Transplant tolerance has been achieved by infusion of CD4^+^CD25^+^ Tregs or CD4^+^Foxp3^+^ Tregs in a variety of animal models (106, 107). CD4^+^Foxp3^+^ Tregs have been studied as a potential therapeutic in human solid organ transplantation with the objective of minimizing the requirement for immunosuppression in transplantation (108–111), and has been proposed as a potential therapeutic strategy in the islet xenotransplant setting (95). *Ex vivo*, polyclonal expanded human CD4^+^CD25^+high^CD127^-/low^ Tregs have been shown to prevent pig islet xenograft rejection in a humanized mouse model, with IL-10 a major contributor to the suppression of the T-cell–mediated antigraft response (112). This was supported by data showing enhanced *in vitro* suppression of human-anti-pig T cell responses by human CD4^+^CD25^+high^CD127^-^ Tregs that were expanded *in vitro* in the presence of pig peripheral blood mononuclear cells (PBMCs) combined with IL-2/Rapamycin and anti-CD3/CD28 magnetic bead stimulation (113, 114). These enhanced human-anti-pig (112, 115) or baboon-anti-pig Tregs proved effective in prevention of islet xenograft rejection in humanized or baboonized mouse models (114).

## Outcome of Cell Therapies Combined With Costimulation Blockade in NHP Models of Islet Xenotransplantation

Recently, with the COVID-19 pandemic and the unprecedented response to vaccine development, we have seen the importance of NHP studies as an essential pre-clinical pathway for the safe introduction of new vaccines and therapeutics. The International Xenotransplantation Association (IXA) provides guidelines that recommend that all potential clinical xenotransplantation related therapies should be evaluated in a NHP model prior to commencing any clinical trial, as these models most closely resemble the human response in term of innate and acquired immune responses in xenotransplantation (116). The rationale for undertaking pre-clinical evaluation in NHP has been to remove uncertainty regarding the safety and clinical benefit prior to early phase clinical trials (117). Whilst NHP models of islet xenotransplantation have been difficult to develop, they provide essential insights into the safety, efficacy, and long-term function of potential clinical therapeutic approaches (116).

As part of this pre-clinical evaluation, NHP models have been used for the development of cell therapies. The most successful have utilised bone marrow cell transplantation (BMT) as part of the strategy to produce transient chimerism across HLA barriers. Using the BMT regimen that produced kidney transplant tolerance these demonstrated the efficacy of tolerance induction in the allotransplant setting by achieving mixed chimerism of donor hematopoietic cells (118). However long-term islet allograft survival was not achieved as the chimerism was only transient in NHP recipients (119). Although mixed chimerism has been achieved in rat to mouse (120) and pig to humanized mouse model (121), macrochimerism in a preclinical pig to NHP setting has not been achieved (122). An alternative strategy has been the use of polyclonal recipient Tregs which have been shown previously to promote extended chimerism in a murine model (123). This has now been extended to NHP, and more recently there have been several publications that have led to the optimisation of the isolation, expansion and activation of Tregs isolated from NHP (124). Whist they have been shown to facilitate long term non-responsiveness in models of allotransplantation, studies of their impact on xenograft survival have been limited (124). In a separate study, infusion of expanded CD4^+^CD25^hi^CD127^-/low^ autologous Tregs, into NHP with well-functioning islet xenografts after anti-CD154 mAb treatment failed to provide graft specific suppression as withdrawal of maintenance immunosuppression led to prompt islet xenograft rejection (125).

There are several NHP models where anti-CD154 mAb-based strategies have been remarkably effective at preventing islet xenograft rejection. However it is not approved for use in humans. As an alternative, anti-CD40 mAb (2C10R4)-based immunosuppression has been evaluated in a pig-to-NHP islet xenotransplantation model. Whilst anti-CD40 mAb was shown to be effective at prolonging porcine islet graft survival, it was not as effective as anti-CD154 mAb, in terms of preventing rejection and early islet loss. Using a cocktail of monoclonal antibodies to inhibit IL-1, IL-6 and TNF at the time of transplant, followed by a maintenance phase with the JAK inhibitor, tofacitinib, plus an anti-BAFF mAb, long-term survival of porcine islet grafts in diabetic NHP has been achieved (126). Whilst all these agents are approved for clinical use, it is unlikely that would ever be used consecutively in a clinical trial. At this point in time, the overall consensus is that although long term survival and function of islet xenografts can be achieved in NHP, the regimens used are not suitable for clinical use. Whilst modulation of the anti-xenograft response using Treg therapy shows promise further development and refinement is required in order to achieve graft specific suppression *in vivo.*

## Outcome of Genetically Modified Pigs as Islet Donors in NHP Models of Islet Xenotransplantation

**Figure 2:**
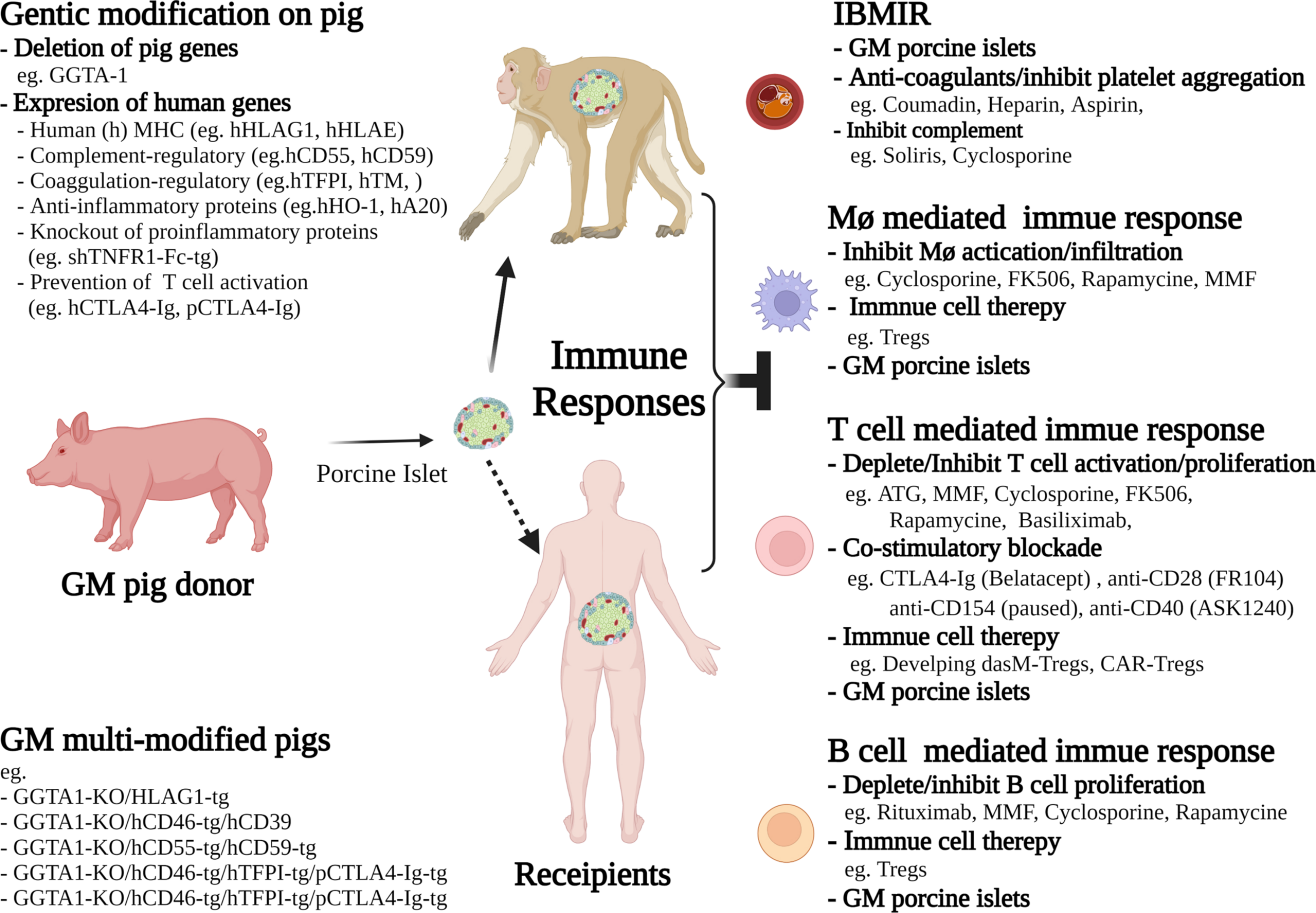
Schematic diagram of selected interventions reported in current non-human primate *(*NHP*) *models, or proposed for future clinical trials in islet xenotransplantation. These current interventions include the strategies of genetic modification (GM) of pig donors, and the therapies for selective inhibition of cellular and humoral immune responses in recipients. The future directions to achieve long-term islet xenograft survival or tolerance include immune cell therapies, such as generating donor antigen specific memory Tregs (dasM-Tregs) or CAR-Tregs for donor antigen specific suppression, and developing advanced immunosuppressive drugs that are more selectively inhibiting and/or depleting effector T cells and B cells as well as suppressing downstream macrophage activation.

To overcome the multiple challenges that are impeding the transition of islet xenotransplantation to the clinic, greater emphasis needs to be placed on the genetic modification of the donor pig with the objective of providing less aggressive treatment of the patient (**Figure 2**). The use of gene editing tools such as CRISPR/Cas9 has meant that it is possible to introduce, or delete, multiple genes in a single somatic cell transfer, which has accelerated the development timeline (127). The issues of hyperacute rejection and IBMIR have largely been resolved by gene modification with the deletion of α-Gal transferase gene, the addition of the human complement regulators, CD46, CD55, CD59 and more recently the addition of the thromboregulatory genes, thrombomodulin and tissue factor pathway inhibitor (9–11). Significant work has been undertaken regarding the development of genetically modified donor pigs that can reduce the cellular xeno-immune response and numerous strategies have been developed to provide the potential for graft tolerance. This includes the expression of Human leukocyte antigen G1 (HLA-G1), a non-classical class I major histocompatibility complex (MHC-I) protein that pays an important role in the maintenance of maternal-fetal tolerance. GKTO/HLAG1+ pigs have been developed and used as islet cell donors with the objective of extending xenograft survival and function in both preclinical NHP models and future clinical trials (128). GTKO/HLAG1+ pigs were shown to modulate the immune response by lowering IFN-γ production by T cells and proliferation of CD4^+^ and CD8^+^ T cells, B cells and NK cells, as well as by augmenting phosphorylation of Src homology region 2 domain-containing phosphatase-2 (SHP-2), which plays a central role in immune suppression. Islets isolated from GTKO/HLA-G1+ genetically engineered pigs and transplanted into streptozotocin-diabetic nude mice restored normoglycemia, suggesting that the expression of HLA-G1 did not interfere with their ability to reverse diabetes (128). Using CRISPR/Cas9, it is possible to target the cellular immune system at multiple levels. Pigs have been generated that express beta-2-microglobulin and HLA-E to inhibit NK cell activation, human CD47 to prevent macrophage induced phagocytosis *via* SIRP-α signalling, as well as the thrombo-regulatory and platelet inhibitory molecules thrombospondin, tissue factor pathway inhibitor and CD39 (11). To reduce the requirements for systemic immunosuppression, other investigators have generated pigs whose islets secrete either CTLA4-Ig or anti-CD2 mAb (129, 130). To date, there have been only a few studies demonstrating limited effectiveness of this strategy in NHP models (9). However, the proof of principle of local immunosuppression has been demonstrated in rodent models (131–133).

## The Way Forward

This review has highlighted the challenges of the cellular immune response in islet xenotransplantation. Currently there is no immunosuppressive strategy that overcomes the dual challenges of efficacy and safety. This brings us back to the need to refocus on one of the major advantages of xenotransplantation; the opportunity to genetically modify the donor with the aim of minimising the treatment required for the recipient. Over the past two decades there have been considerable progress in developing a genetically modified pig islets that overcomes the dual problems of IBMIR and cell mediated rejection. This has been accelerated more recently by the generation of multi-transgenic pigs using CRISPR/Cas9 technology. This new generation of pigs have been designed to target simultaneously several components of the immune system. They have yet to be formally tested in NHP models of islet xenotransplantation, although preliminary studies of porcine kidneys from these pigs transplanted into cynomologus monkeys have shown promising results (134). A new option for islet cell replacement has been reported recently. In February of 2022 ViaCyte/CRISPR announced in a press release that their first patient was treated in phase 1 trial of gene-edited islet cell replacement therapy for type1 diabetes, suggesting the development of gene-edited, stem cell-derived, “hypoimmunogenic” islet cell products as a promising alternative strategy for treatment of T1D. Whilst this sounds promising, formal evaluation of the trial results in the peer reviewed literature is awaited. As with many new medical immunosuppressive reagents, progress in xenotransplantation has been governed by technological advances and the “next generation” of genetically modified pigs could result in porcine islet xenotransplantation being a successful treatment for T1D.

## Author Contributions

PO coordinated the planned the article, wrote, edited and reviewed the contents. MH, WH, and SY wrote, and reviewed content. All authors contributed to the article and approved the submitted version.

## Funding

Work undertaken by the authors was supported by grants from the National Health and Medical Research Council of Australia and the Juvenile Diabetes Research Foundation. POC was supported by an NHMRC Senior Practitioner Fellowship.

## Conflict of Interest

The authors declare that the research was conducted in the absence of any commercial or financial relationships that could be construed as a potential conflict of interest.

## Publisher’s Note

All claims expressed in this article are solely those of the authors and do not necessarily represent those of their affiliated organizations, or those of the publisher, the editors and the reviewers. Any product that may be evaluated in this article, or claim that may be made by its manufacturer, is not guaranteed or endorsed by the publisher.
